# A head-to-head comparison of the intra- and interobserver agreement of COVID-RADS and CO-RADS grading systems in a population with high estimated prevalence of COVID-19

**DOI:** 10.1259/bjro.20200053

**Published:** 2020-12-11

**Authors:** Nikita Sushentsev, Vlad Bura, Maruša Kotnik, Grigoriy Shiryaev, Iztok Caglic, Jonathan Weir-McCall, Tristan Barrett

**Affiliations:** 1 Department of Radiology, Addenbrooke’s Hospital and University of Cambridge, Cambridge, United Kingdom; 2 Department of Radiology, County Clinical Emergency Hospital, Cluj-Napoca, Romania; 3 Royal Papworth Hospital NHS Foundation Trust, Cambridge, United Kingdom; 4 COVID-19 Center, National Medical Research Center of Cardiology, Moscow, Russian Federation, Russia

## Abstract

**Objective:**

To evaluate the inter- and intraobserver agreement of COVID-RADS and CO-RADS reporting systems among differently experienced radiologists in a population with high estimated prevalence of COVID-19.

**Methods and materials:**

Chest CT scans of patients with clinically–epidemiologically diagnosed COVID-19 were retrieved from an open-source MosMedData data set, randomised, and independently assigned COVID-RADS and CO-RADS grades by an abdominal radiology fellow, thoracic imaging fellow and a consultant cardiothoracic radiologist. The inter- and intraobserver agreement of the two systems were assessed using the Fleiss’ and Cohen’s κ coefficients, respectively.

**Results:**

A total of 200 studies were included in the analysis. Both systems demonstrated moderate interobserver agreement, with κ values of 0.51 [95% confidence interval (CI): 0.46–0.56] and 0.55 (95% CI: 0.50–0.59) for COVID-RADS and CO-RADS, respectively. When COVID-RADS and CO-RADS grades were dichotomised at cut-off values of 2B and 4 to evaluate the agreement between grades representing different levels of clinical suspicion for COVID-19, the interobserver agreement became substantial with κ values of 0.74 (95% CI: 0.66–0.82) for COVID-RADS and 0.73 (95% CI: 0.65–0.81) for CO-RADS. The median intraobserver agreement was considerably higher for CO-RADS reaching 0.81 (95% CI: 0.43–0.76) compared with 0.60 (95% CI: 0.43–0.76) of COVID-RADS.

**Conclusions:**

COVID-RADS and CO-RADS showed comparable interobserver agreement, which was moderate when grades were compared head-to-head and substantial when grades were dichotomised to better reflect the underlying levels of suspicion for COVID-19. The median intraobserver agreement of CO-RADS was, however, considerably higher compared with COVID-RADS.

**Advances in knowledge:**

This paper provides a comprehensive review of the newly introduced COVID-19 chest CT reporting systems, which will help radiologists of all sub-specialties and experience levels make an informed decision on which system to use in their own practice.

## Introduction

According to the latest WHO figures, the COVID-19 pandemic caused by severe acute respiratory syndrome coronavirus 2 (SARS-CoV-2) has been associated with more than 25,000,000 confirmed cases and 848,000 deaths worldwide.^1^ A series of unprecedented large-scale non-pharmaceutical interventions introduced across Europe has reportedly led to 3,100,000 deaths being averted.^2^ Despite the gradual easing of lockdown measures, health-care services across Europe remain under continuous pressure with radiology departments remaining on the frontline of the COVID-19 diagnostic pathway.^3–5^

Although major international institutions, including the British Society of Thoracic Imaging (BSTI) and the American College of Radiology, argue against the routine use of chest CT for diagnosis and triage of patients with suspected COVID-19, the advantage of unenhanced chest CT over real-time polymerase chain reaction (RT-PCR) as a rapid and prognostically valuable first-line investigation in symptomatic and comorbid patients, particularly in high prevalence areas,has been highlighted in several studies.^6–11^ The recent multinational consensus statement from the Fleischner Society, however, highlights the greater sensitivity of chest CT to early pneumonic changes compared to chest X-ray and acknowledges the preferred use of the former modality in severely affected areas, where the reliability of RT-PCR testing is limited and turnaround times are long.^12^ Although an extensive body of literature has emerged describing characteristic CT features of COVID-19 at different stages of the disease, considerable differences in reporting practices have been highlighted.^13–16^ With repeated waves of the pandemic being forecast,^2^ radiologists of all experience levels and subspecialties will be expected to contribute to the effective triage of patients with suspected COVID-19. To ensure optimal results, this requires the development of standardised reporting systems with high intra- and interobserver agreement.

With this in mind, two grading systems for standardised assessment of unenhanced chest CT in patients with suspected COVID-19 were independently proposed in late April 2020: CO-RADS and COVID-RADS.^17,18^ COVID-RADS represents a 5-point scale with a supporting lexicon that clearly defines specific findings one needs to observe in order to assign a score indicating low, moderate, and high suspicion level of COVID-19 pneumonia.In contrast to providing a list of specific features needed to assign each individual grade, CO-RADS combines them in patterns indicating five different levels of suspicion ranging from very low to very high, also incorporating grades 0 and 6 that are assigned when a study is of insufficient quality or accompanied by a positive RT-PCR test. CO-RADS has been internally validated against RT-PCR (AUC 0.91, 95% CI 0.85–0.97) and its interobserver agreement among eight radiologists with different experience in reading chest CTs accounted for a Fleiss’ κ of 0.47 (95% CI 0.45–0.49).^17^ Conversely, there are no published reports of a similar validation of COVID-RADS, and no attempts have been made to conduct a direct comparison between the two systems when applied to a population with high estimated COVID-19 prevalence by radiologists with different experience levels.

In this study, chest CT scans of patients with clinical–epidemiological diagnosis of COVID-19 were reviewed by differently experienced radiologists from three European countries with the objective of comparing the intra- and interobserver agreement of COVID-RADS and CO-RADS grading systems in ahigh prevalence setting.

## Methods

### Data set description

In this retrospective study, we used anonymised unenhanced chest CT images obtained from the open-source *MosMedData* data set published by the Research and Practical Clinical Center for Diagnostics and Telemedicine Technologies of the Moscow Health Care Department. The data set includes 1110 individual chest CT studies (slice thickness 1.0–1.5 mm) of patients with clinical–epidemiological diagnosis of COVID-19 (ICD-10 code U07.2) performed in municipal hospitals in Moscow, Russian Federation, between 1 March and 25 April 2020. The studies, stored in the NifTI format, were categorised by the authors of the data set into five groups depending on the degree of pulmonary involvement, ranging from scans representing normal chest (CT-0) to those with detected ground glass opacifications (GGOs), regions of consolidation, reticular changes and hydrothorax with more than 75% of lung tissue involved (CT-4) as per Russian national guidelines. The data set is licensed under a Creative Commons Attribution-NonCommercial-NoDerivs 3.0 Unported (CC BY-NC-ND 3.0) license and is available via a permanent link https://mosmed.ai/datasets/covid19_1110.

### Patient selection process

**Figure 1.**
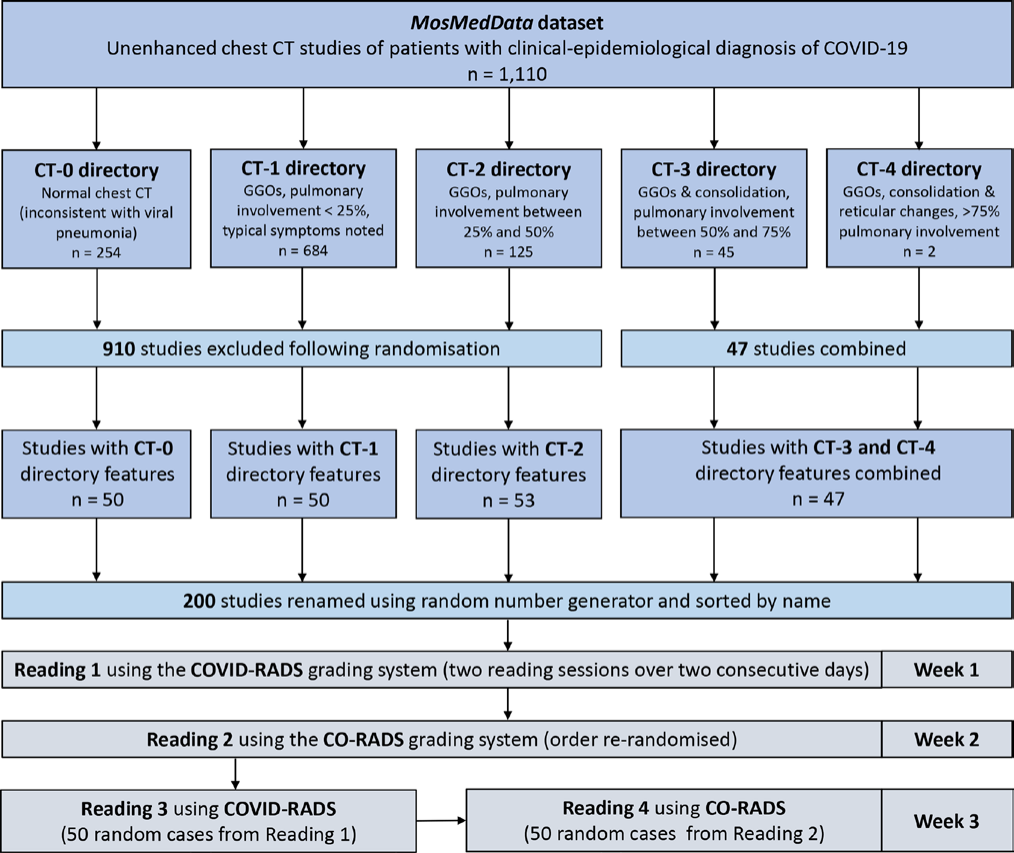
Flow diagram of patient selection and reading process.

To maximise the use of the available imaging data while also ensuring adequate distribution of studies with different predefined degrees of pulmonary involvement, all cases from the CT-3 and CT-4 groups were combined and matched by randomly selected studies from the remaining three groups CT-0, CT-1 and CT-2 to produce an overall proportion of 1:1:1:1 with a total sample size of 200 studies, the order of which was then randomized (Figure 1).

### Chest CT interpretation using COVID-RADS and CO-RADS grading systems

Three radiologists from different tertiary referral centres from three different countries independently reviewed the studies and assigned COVID-RADS and CO-RADS scores using the originally described scoring systems as a reference (Supplementary Material 1).^16,17^ Given the nature of the selected data set, there were no studies for which CO-RADS grades 0 (not interpretable) or 6 (RT-PCR positive for SARS-CoV-2) could be assigned. Although the readers were aware of the clinical–epidemiological diagnosis of COVID-19 in the selected patients, they were blinded to the predefined CT category. Before completing the reading process as illustrated in Figure 1, all readers completed a training set of cases that they had selected randomly from the original data set. The first two readings, the outcomes of which were used to calculate the interobserver agreement of COVID-RADS and CO-RADS, respectively, took place within a week from one another with cases being re-randomised for the second reading. Readings 3 and 4, which took place within a week of reading 2, were used to calculate the intraobserver agreement of the two systems and each consisted of 50 re-randomised cases. The images were viewed using an open-source software ITK-SNAP, with readers being able to modify the window settings.^19^ Reader 1 (VB) worked at an emergency medicine hospital and was a senior abdominal radiology fellow with no experience of routine reporting chest CTs of patients with suspected COVID-19. Reader 2 (MK) was a thoracic imaging fellow at a large regional heart and lung hospital with 4 years’ overall experience reporting chest CTs and 3 months’ experience reporting chest CTs of patients with suspected COVID-19. Reader 3 (GS) was a consultant cardiothoracic radiologist at a regional COVID-19 referral centre with 11 years’ experience of reporting chest CTs.

### Statistics

The outcomes of Readings 1 and 2 were analysed to calculate the interobserver agreement of COVID-RADS and CO-RADS grading systems among the three readers using the Fleiss’ κ with 95% confidence intervals (CIs). The analysis was repeated after COVID-RADS and CO-RADS scores were dichotomised at cut-off values of 2A and 3, respectively, to evaluate the interobserver agreement between the COVID-19 levels of suspicion that may directly impact clinical decision-making (low and moderate for COVID-RADS *vs* equivocal, high and very high suspicion for CO-RADS). Additional dichotomisation was performed at cut-off values of 2B and 4 in order to assess the agreement between the grades that include typical COVID-19 features and therefore represent the highest levels of clinical suspicion the two systems can offer. Linearly weighted Cohen’s κ was calculated to assess the interobserver agreement between individual readers. The intraobserver agreement of COVID-RADS and CO-RADS was calculated using Cohen’s simple κ using the outcomes of Readings 3 and 4, respectively.^20^ The κ values were interpreted as follows: values ≤ 0 as indicating less than chance agreement, 0.01–0.20 as slight, 0.21–0.40 as fair, 0.41–0.60 as moderate, 0.61–0.80 as substantial and 0.81–0.99 as almost perfect agreement.

## Results

Of the 1110 chest scans included in the original data set, 466 (42%) were male patients, 622 (56%) female patients and 22 (2%) were patients whose gender was unknown. Median age was 47 years (range 18–97 years).

### Interobserver agreement of COVID-RADS and CO-RADS grading systems

For COVID-RADS and CO-RADS, the Fleiss’ κ values were 0.51 (95% CI: 0.46–0.56) and 0.55 (95% CI: 0.50–0.59), respectively, indicating moderate agreement for both systems, with κ values for individual scores presented in Table 1. Following dichotomisation at cut-off values of 2A and 3 (Table 2), the Fleiss’ κ values for COVID-RADS and CO-RADS increased up to 0.77 (95% CI: 0.69 to 0.85) and 0.81 (95% CI: 0.73–0.89), falling into substantial and almost perfect interobserver agreement categories, respectively. When the grades were dichotomised at cut-off values of 2B and 4 (Table 2), the Fleiss’ κ values were 0.74 (95% CI: 0.66–0.82) and 0.73 (95% CI: 0.65–0.81) for COVID-RADS and CO-RADS, respectively.

**Table 1.** Fleiss’ κ values with 95% CIs demonstrating the interobserver agreement of each individual COVID-RADS and CO-RADS grade

Grade	Overall Fleiss' κ	95% CI
COVID-RADS 0	0.63	0.55–0.71
COVID-RADS 1	0.15	0.07–0.23
COVID-RADS 2A	0.13	0.05–0.21
COVID-RADS 2B	0.31	0.23–0.39
COVID-RADS 3	0.68	0.60–0.76
CO-RADS 1	0.84	0.76–0.92
CO-RADS 2	0.39	0.31–0.47
CO-RADS 3	0.26	0.18–0.34
CO-RADS 4	0.35	0.28–0.43
CO-RADS 5	0.58	0.50–0.66

CI, confidence interval.

**Table 2.** Fleiss’ κ values with 95% CIs demonstrating the interobserver agreement of COVID-RADS and CO-RADS grades, grouped based on the underlying level of suspicion for COVID-19 (with cut-off scores of 2A and 3, respectively) and the presence of typical/compulsory COVID-19 features (with cut-off values of 2B and 4, respectively)

COVID-19 level of suspicion	Grades grouped	Fleiss' κ values (95% CI)
Low	COVID-RADS 0 and 1	0.83 (0.75–0.91)
Moderate to high	COVID-RADS 2A, 2B, 3	0.94 (0.86–1.02)
	**Overall**	**0.77 (0.69–0.85**)
Very low to low	CO-RADS 1 and 2	0.86 (0.78–0.94)
Equivocal to very high	CO-RADS 3, 4 and 5	0.95 (0.87–1.03)
	**Overall**	**0.81 (0.73–0.89**)
Low to moderate*	COVID-RADS 0, 1 and 2A	0.82 (0.76–0.90)
Moderate to high*	COVID-RADS 2B and 3	0.92 (0.84–1.00)
	**Overall**	**0.74 (0.66–0.82**)
Very low to equivocal	CO-RADS 1, 2 and 3	0.83 (0.75–0.91)
High to very high	CO-RADS 4 and 5	0.90 (0.82–0.98)
	**Overall**	**0.73 (0.65–0.81**)

CI, confidence interval.

At an individual level, the agreement of COVID-RADS was highest between Readers 1 and 2 and lowest between Readers 2 and 3. For CO-RADS, the highest agreement was again observed between Readers 1 and 2 and the lowest agreement was noted between Readers 1 and 3 (Table 3).

**Table 3.** Interobserver agreement of COVID-RADS and CO-RADS between individual readers

Reader pair	COVID-RADS agreement	CO-RADS agreement
	Fleiss' κ (95% CI)	Fleiss' κ (95% CI)
**Reader 1–Reader 2**	0.82 (0.77–0.87)	0.77 (0.71 *vs* 0.83)
**Reader 1–Reader 3**	0.67 (0.58–0.75)	0.70 (0.62–0.78)
**Reader 2–Reader 3**	0.66 (0.58–0.74)	0.73 (0.66–0.79)

CI, confidence interval.

**Figure 2.**
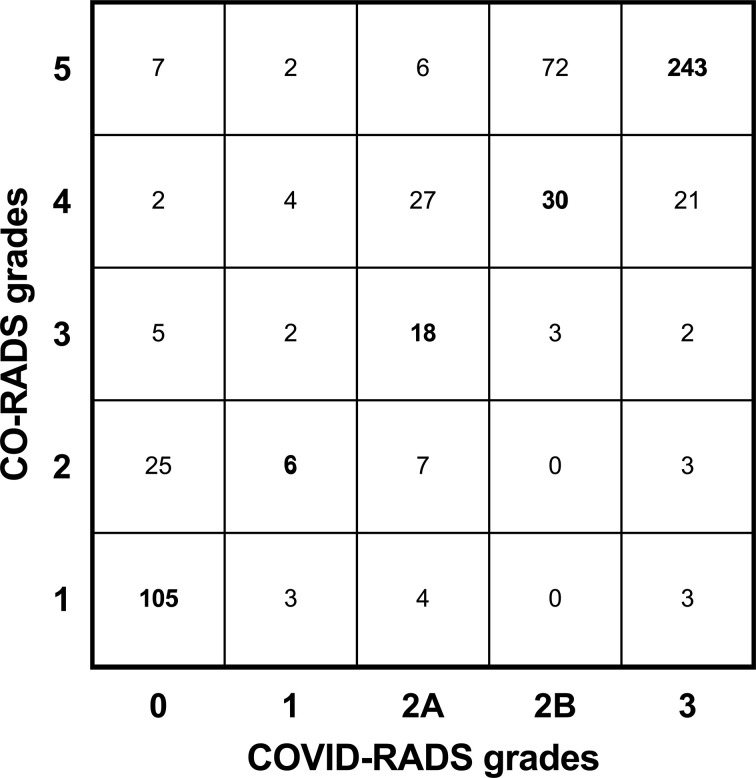
A confusion matrix illustrating the agreement between individual COVID-RADS and CO-RADS grades.

The outcomes of a direct comparison of the interobserver agreement of individual COVID-RADS and CO-RADS grades is illustrated in Figure 2. Despite the intrinsic differences in the definition of each grade, the two systems showed a moderate interobserver agreement with the Cohen’s κ of 0.51 (95% CI: 0.46–0.56).

### Discrepant cases

**Figure 3.**
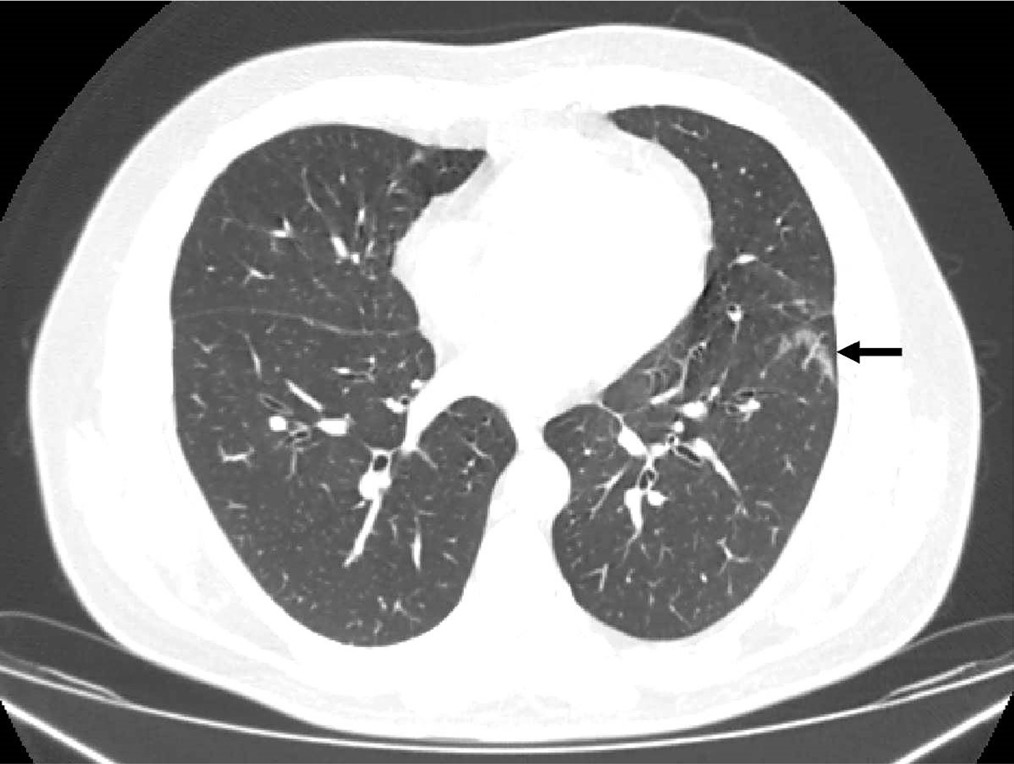
An example double-grade discrepant case for COVID-RADS. In the absence of other abnormal findings, Readers 1 and 2 considered the abnormality (black arrow) as a single GGO and therefore called it COVID-RADS 2A and CO-RADS 3, implying equivocal and moderate levels of suspicion for COVID-19, respectively. Reader 3, however, considered this as an area of fibrosis and called it COVID-RADS 0 and CO-RADS 1, thereby giving this case a very low level of suspicion. GGO, ground glass opacification.

Absolute agreement in COVID-RADS grades was detected in 114/200 (57%) cases. Single-grade discrepancies were noted in 74/200 (37%) cases, of which discrepancies between grades 2B and 3 were the most frequent (34/74; 46%). Double-grade discrepancies were observed in 10/200 cases (5%), of which 8/10 (80%) were between COVID-RADS grades 0 and 2A (Figure 3) or 1 and 2B. In 13/200 (7%) cases, discrepancies occurred between either COVID-RADS grades 0 and 3 (6/13; 46%) or 1 and 3 (7/13; 54%).

**Figure 4.**
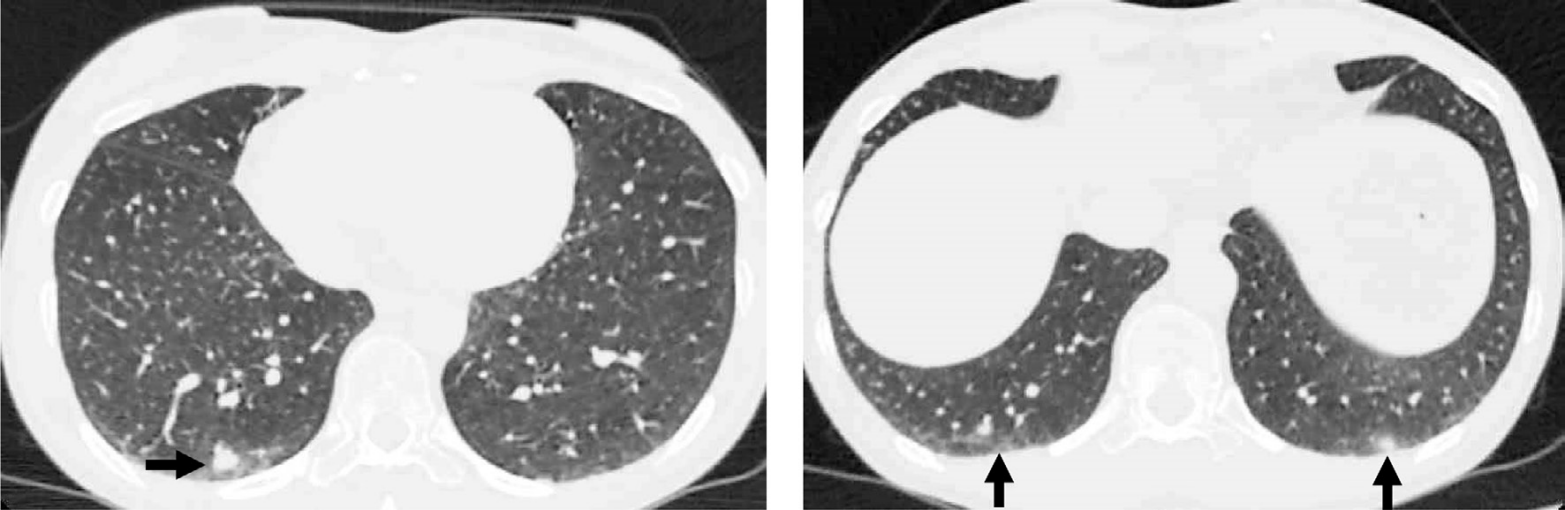
An example single-grade discrepant case between COVID-RADS and CO-RADS. All readers noted multiple bilateral GGOs (black arrows) located posteriorly at lung bases in the presence of a marked nodular pattern, which gave this case an atypical appearance. Multiple GGOs located close to the visceral pleura represent a mandatory feature of CO-RADS 5, automatically leading to the highest level of suspicion for COVID-19 without the opportunity to somehow highlight their atypical nodular appearance. However, COVID-RADS specifically lists nodular pattern as an atypical finding (Grade 1) that in combination with multiple GGOs (Grade 3) comprises a combined score of 2B, providing readers with an opportunity to highlight cases where certain combinations may slightly reduce the overall level of suspicion for COVID-19. GGO, ground glass opacification.

For CO-RADS scores, absolute agreement was observed in 115/200 (58%) cases. Single-grade discrepancies represented 60/200 (30%) cases, of which 48/60 (80%) were between CO-RADS grades 4 and 5. Double-grade discrepancies accounted for 34/200 (17%), of them 23/34 (68%) being between CO-RADS scores 3 and 5 (Figure 4). In 10/200 (5%) cases, discrepancies occurred between CO-RADS grades 2 and 5 (5/10; 50%), 1 and 5 (1/10; 10%) and 0 and 5 (4/10; 40%).

An example case where clinically significant discrepancy was observed in both COVID-RADS and CO-RADS is presented in Supplementary Material 1.

### Intraobserver agreement of COVID-RADS and CO-RADS grading systems

The median intraobserver agreement of COVID-RADS among the three readers was 0.60 (95% CI: 0.43–0.76) whilst CO-RADS demonstrated a considerably higher median intraobserver agreement of 0.81 (95% CI: 0.66–0.95). Individual Cohen’s κ values for the three readers are presented in Table 4. At an intraobserver level, 60 and 47% of discrepancies occurred between COVID-RADS Grades 2B and three and CO-RADS Grades 4 and 5, respectively.

**Table 4.** Cohen’s κ with 95% CIs demonstrating the intraobserver agreement of COVID-RADS and CO-RADS grades for Reader 1 (abdominal radiology fellow), Reader 2 (thoracic imaging fellow) and Reader 3 (cardiothoracic radiologist)

Reader	COVID-RADS intraobserver agreement	CO-RADS intraobserver agreement
	Cohen’s κ (95% CI)	Cohen’s κ (95% CI)
**Reader 1**	0.86 (0.74–0.99)	0.93 (0.84–1.02)
**Reader 2**	0.53 (0.37–0.68)	0.81 (0.66–0.95)
**Reader 3**	0.60 (0.43–0.76)	0.74 (0.58–0.91)

CI, confidence interval.

### Distribution of COVID-RADS and CO-RADS grades among patients with different degree of pulmonary involvement

The distribution of COVID-RADS and CO-RADS grades among patients with different pre-defined CT features is summarised in Supplementary Material 1, respectively. On average, 91% of both COVID-RADS grades 0 and CO-RADS grades 1 were assigned by the readers to CT-0 directory studies, defined by the authors of the data set as containing CTs inconsistent with viral pneumonia including COVID-19. Conversely, COVID-RADS grades 3 and CO-RADS grades 5 (and to a slightly lesser extent 2B and 4) were proportionately distributed by the three readers among studies from CT-1, CT-2 and CT-3/4 directories, suggesting equal occurrence of findings consistent with COVID-19 regardless of the overall degree of pulmonary involvement.

## Discussion

In this study, three differently experienced radiologists from different countries independently assigned COVID-RADS and CO-RADS grades to 200 chest CTs of patients with clinically–epidemiologically diagnosed COVID-19. When applied to a population with high pre-test probability of COVID-19, COVID-RADS and CO-RADS demonstrated comparable interobserver agreement with Fleiss’ κ values of 0.51 and 0.55, respectively. However, the median intraobserver agreement was considerably higher for CO-RADS.

In this study, the overall interobserver agreement of CO-RADS was broadly in agreement with that reported originally by Prokop et al. (0.55 *vs* 0.47), with slight differences expected due to the differences in the study populations and the number of readers.^17^ The agreement between individual readers was also in line with that recently reported by de Jaegere et al.^21^ The marginally higher agreement of CO-RADS may be explained by several intrinsic differences between the two systems that are clearly visible when κ values of individual scores are compared with each other. It is of note that COVID-RADS had higher interobserver agreement between less experienced readers, which might be explained by its “feature-centric” nature rather than a “pattern-centric” structure of CO-RADS. In other words, COVID-RADS provides a well-defined lexicon of specific features, combinations of which comprise individual scores, thereby offering a more structured approach that may be more appreciated by less experienced readers. Conversely, CO-RADS provides higher flexibility that makes it easier to account for the “bigger picture,” thereby requiring a certain degree of experience to be used confidently. This point can be supported by higher interobserver agreement of CO-RADS between more experienced readers that was reported in this study. Finally, in contrast to a subtle difference in the overall interobserver agreement between the two systems, intraobserver agreement of CO-RADS was considerably higher for all readers. A possible explanation is that once readers get used to the patterns described in CO-RADS, their subsequent use then becomes less dependent on subtle features that make more difference for the “feature-centric” COVID-RADS.

There are certain characteristic features of each system that require particular attention. CO-RADS 1 combines features consistent with both normal findings and those of unequivocal non-infectious aetiology, which in COVID-RADS are represented by two different grades, 0 and 1, likely contributing to its lower intra- and interobserver agreement. Furthermore, CO-RADS 3 includes GGOs that do not have an appearance typical for COVID-19, *e.g*. perihilar GGOs.^22^ In contrast, COVID-RADS 2A allows for the presence of only a single area of GGO, whereas peribronchovascular GGOs fall into the COVID-RADS 1 category, again providing the basis for some clinically significant interobserver variation since COVID-RADS 1 and 2A imply different levels of suspicion for COVID-19. Furthermore, the flexibility of CO-RADS 3 in relation to the number and localisation of GGOs makes it easier to be assigned in cases when false-positive GGOs related to motion, hypoventilation or air trapping are suspected (as illustrated in Figure 3). Conversely, COVID-RADS has a clearer definition of typical findings (score 3) that in CO-RADS are more cautiously distributed between scores 4 and 5, *e.g.* making a distinction between unilaterally or bilaterally located GGOs, thereby leading to an overlap with COVID-RADS grades 2A, 2B and three as evidenced in Figure 2. In addition to the higher κ for COVID-RADS 3 compared to CO-RADS 5, this trend is clearly evidenced by the fact that the overwhelming majority of single-grade discrepancies of CO-RADS occurred between scores 4 and 5, resulting in a substantial improvement in the interobserver agreement of the two systems when scores were dichotomised at cut-off levels of 2B and 4. It should also be stressed that neither system takes into account the overall degree of pulmonary involvement, which was confirmed by the equal distribution of studies with the highest COVID-RADS and CO-RADS grades among patients with different predefined CT groups. As illustrated in Figure 4, another potential benefit of COVID-RADS is the presence of score 2B that allows to highlight cases where typical COVID-19 features are mixed with atypical findings. This core difference between the two systems is further reflected in Figure 2, where 72 CO-RADS 5 cases were assigned COVID-RADS 2B. It is of note, however, that the majority of single-grade discrepancies of COVID-RADS occurred between scores representing different levels of clinical suspicion of COVID-19 (0 *vs* 2A and 1 *vs* 2B), which may warrant updating the definition of these scores in order to avoid potentially clinically-significant discrepancies.

This study has several limitations. Validation of the two grading systems against RT-PCR was not possible due to the unavailability of this information in the original data set, however, evaluating the diagnostic utility of COVID-RADS and CO-RADS was not the aim of this study. However, as pointed out by the authors of both systems, they are primarily applicable to epidemic areas with high estimated prevalence of the disease, and were themselves developed in high-prevalence settings, in which CT allows for a faster and more accurate triage of patients at initial presentation.^17,18^ Furthermore, as pointed out by Chen et al, long detection time and dependence on adequate sampling make RT-PCR less adaptable to the clinical workflow and decision-making during an outbreak, implying the need to isolate patients with positive CT findings even in the presence of a negative RT-PCR.^15^ In addition, two recently published studies investigating the diagnostic accuracy of CO-RADS in RT-PCR confirmed cohorts confirmed its good performance in symptomatic individuals, thereby further supporting its application for triage.^23,24^ Moreover, the readers were not blinded to the presence of clinical diagnosis of COVID-19 in the included patients, which may have artificially increased the interobserver agreement of the two systems due to the introduced bias. This, however, is also representative of a real-life clinical scenario during an epidemiologically severe situation where high pretest prevalence of COVID-19 is estimated and supporting clinical information is almost always available to the reporting clinicians, which was the case in a recent study evaluating the agreement of the RSNA COVID-19 chest CT classification scheme.^21^ Furthermore, high vigilance for COVID-19 is likely to remain even between the repeated waves of the epidemic, which makes a certain degree of bias inevitable.Finally, clinical suspicion for COVID-19, which is essentially a clinical–epidemiological diagnosis, is imperative for requesting imaging studies in real-life practice as mandated by the BSTI guidelines.^25^ The reading sessions were relatively close together in time, which may have increased the reported intraobserver agreement. The distribution of cases with predefined CT groups in the study group differed considerably from the original dataset, however, this was done to avoid bias associated with over representation of CT-1 group (<25% pulmonary involvement) that could have artificially increased both intra- and interobserver agreement reported in this study. Moreover, there were no studies with CO-RADS scores 0 and 6, however, this did not make the data set non-comparable against COVID-RADS. In turn, the presence of scores 0 and 6 could artificially increase the κ values for CO-RADS as these scores are likely to have almost perfect interobserver agreement by their nature. Finally, we acknowledge the recent development of other standardised reporting systems such as the aforementioned RSNA scheme, chest CT patterns of BSTI guidelines, COVID-19 S, etc that all have their comparative advantages, evaluating which, however, is beyond the scope of this study.^21,25,26^

In conclusion, both COVID-RADS and CO-RADS represent reproducible reporting systems of chest CTs of patients with suspected COVID-19 and can be confidently used by differently experienced radiologists in a population with high estimated prevalence of the disease, which is further supported by other studies suggesting high diagnostic performance of the two systems compared to RT-PCR testing. Whilst CO-RADS has a considerably higher intraobserver agreement and a slightly higher overall interobserver agreement across all readers, this is counterbalanced by a higher interobserver agreement of COVID-RADS between less experienced readers and a similar interobserver agreement when it comes to scores representing high clinical suspicion of the disease, thereby suggesting possible interchangeability of the two systems depending on the individual reader’s preferences and experience level, with factors informing the final choice summarised in this study.

## References

[1] WHO Coronavirus Disease (COVID-19) Dashboard.. Available from: https://covid19.who.int/?gclid=Cj0KCQjwzZj2BRDVARIsABs3l9JhSWgAXnkDblcWewYS30t3L85C88E_FGOMDoBG0YbqNpGBnVCEissaAooxEALw_wcB [Accessed May 21, 2020].

[2] Flaxman S, Mishra S, Gandy A, Unwin HJT, Mellan TA, Coupland H, et al Estimating the effects of non-pharmaceutical interventions on COVID-19 in Europe. Nature 2020; 584: 257–61. doi: 10.1038/s41586-020-2405-7 32512579

[3] Kim H Outbreak of novel coronavirus (COVID-19): what is the role of radiologists? Eur Radiol 2020; 30: 3266–7. doi: 10.1007/s00330-020-06748-2 32072255 PMC7087878

[4] Chen RC, Tan TT, Chan LP Adapting to a new normal? 5 key operational principles for a radiology service facing the COVID-19 pandemic. European Radiology 2020;: 1–4. 32328761 10.1007/s00330-020-06862-1 PMC7178921

[5] Politi LS, Balzarini L The radiology department during the COVID-19 pandemic: a challenging, radical change. Eur Radiol 2020; 30: 3600–2. doi: 10.1007/s00330-020-06871-0 32318843 PMC7170792

[6] Fang Y, Zhang H, Xie J, Lin M, Ying L, Pang P, et al Sensitivity of chest CT for COVID-19: comparison to RT-PCR. Radiology 2020; 296: E115–7. doi: 10.1148/radiol.2020200432 32073353 PMC7233365

[7] Ai T, Yang Z, Hou H, Zhan C, Chen C, Lv W, et al Correlation of chest CT and RT-PCR testing for coronavirus disease 2019 (COVID-19) in China: a report of 1014 cases. Radiology 2020; 296: E32–40. doi: 10.1148/radiol.2020200642 32101510 PMC7233399

[8] Revel MP, Parkar AP, Prosch H, et al COVID-19 patients and the radiology department – advice from the European Society of radiology (ESR) and the European Society of thoracic imaging (ESTI. Eur Radiol 2020;: 1–7. 32314058 10.1007/s00330-020-06865-y PMC7170031

[9] Long C, Xu H, Shen Q, Zhang X, Fan B, Wang C, et al Diagnosis of the coronavirus disease (COVID-19): rRT-PCR or CT? Eur J Radiol 2020; 126: 108961. Epub ahead of print May 1, 2020. doi: 10.1016/j.ejrad.2020.108961 32229322 PMC7102545

[10] Watson J Interpreting a covid-19 test result.. 10.1136/bmj.m1808 32398230

[11] Wynants L, Van Calster B, Collins GS, Riley RD, Heinze G, Schuit E, Bonten MMJ, et al Prediction models for diagnosis and prognosis of covid-19 infection: systematic review and critical appraisal. BMJ 2020; 369: m1328. Epub ahead of print April 7, 2020. doi: 10.1136/bmj.m1328 32265220 PMC7222643

[12] Rubin GD, Haramati LB, Kanne JP, et al The role of chest imaging in patient management during the COVID-19 pandemic: a multinational consensus statement from the Fleischner Society. Radiology 2020; 201365. 10.1148/radiol.2020201365 PMC7233395 32255413

[13] Salehi S, Abedi A, Balakrishnan S, Gholamrezanezhad A Coronavirus disease 2019 (COVID-19): a systematic review of imaging findings in 919 patients. AJR Am J Roentgenol 2020; 215: 1–7. doi: 10.2214/AJR.20.23034 32174129

[14] Bao C, Liu X, Zhang H, Li Y, Liu J Coronavirus disease 2019 (COVID-19) CT findings: a systematic review and meta-analysis. J Am Coll Radiol 2020; 17: 701–9. Epub ahead of print March 25, 2020. doi: 10.1016/j.jacr.2020.03.006 32283052 PMC7151282

[15] Chen HJ, Qiu J, Wu B, et al Early chest CT features of patients with 2019 novel coronavirus (COVID-19) pneumonia: relationship to diagnosis and prognosis.. 10.1007/s00330-020-06978-4 PMC7280678 32518987

[16] Ojha V, Mani A, Pandey NN, Sharma S, Kumar S, et al Ct in coronavirus disease 2019 (COVID-19): a systematic review of chest CT findings in 4410 adult patients. Eur Radiol 382. doi: 10.1007/s00330-020-06975-7 PMC7261039 32474632

[17] Prokop M, van Everdingen W, van Rees Vellinga T, Quarles van Ufford H, Stöger L, Beenen L, et al CO-RADS: a categorical CT assessment scheme for patients suspected of having COVID-19-Definition and evaluation. Radiology 2020; 296: E97–104 Epub ahead of print. doi: 10.1148/radiol.2020201473 32339082 PMC7233402

[18] Salehi S, Abedi A, Balakrishnan S, et al Coronavirus disease 2019 (COVID-19) imaging reporting and data system (COVID-RADS) and common lexicon: a proposal based on the imaging data of 37 studies. Eur Radiol 2020;: 1–13. 32346790 10.1007/s00330-020-06863-0 PMC7186323

[19] Yushkevich PA, Piven J, Hazlett HC, Smith RG, Ho S, Gee JC, et al User-guided 3D active contour segmentation of anatomical structures: significantly improved efficiency and reliability. Neuroimage 2006; 31: 1116–28. doi: 10.1016/j.neuroimage.2006.01.015 16545965

[20] Gwet KL Intrarater Reliability : Hoboken N. J, Wiley Encyclopedia of Clinical Trials. USA: John Wiley & Sons, Inc; 2008 1–13.

[21] de Jaegere TMH, Krdzalic J, Fasen BACM, Kwee RM, et al.COVID-19 CT Investigators South-East Netherlands (CISEN) study group Radiological Society of North America chest CT classification system for reporting COVID-19 pneumonia: interobserver variability and correlation with RT-PCR. Radiology 2020; 2: e200213. doi: 10.1148/ryct.2020200213 PMC7294823 33778589

[22] Simpson S, Kay FU, Abbara S, Bhalla S, Chung JH, Chung M, et al Radiological Society of North America expert consensus statement on reporting chest CT findings related to COVID-19. endorsed by the Society of thoracic radiology, the American College of radiology, and RSNA. Radiology 2020; 2: e200152. doi: 10.1148/ryct.2020200152 PMC7233447 33778571

[23] De Smet K, De Smet D, Ryckaert T, et al Diagnostic performance of chest CT for SARS-CoV-2 infection in individuals with or without COVID-19 symptoms. Radiology 2020; 202708. 10.1148/radiol.2020202708 PMC7418927 32776832

[24] Fujioka T, Takahashi M, Mori M, Tsuchiya J, Yamaga E, Horii T, et al Evaluation of the usefulness of CO-RADS for chest CT in patients suspected of having COVID-19. Diagnostics 2020; 10: 608. doi: 10.3390/diagnostics10090608 32825060 PMC7555303

[25] British Society of Thoracic Imaging Thoracic Imaging in COVID-19 Infection: Guidance for the Reporting Radiologist. 2020 Available from: https://www.bsti.org.uk/media/resources/files/BSTI_COVID-19_Radiology_Guidance_version_2_16.03.20.pdf [Accessed September 2, 2020].

[26] Gezer NS, Ergan B, Barış MM, Appak Özgür, Sayıner AA, Balcı P, et al COVID-19 S: a new proposal for diagnosis and structured reporting of COVID-19 on computed tomography imaging. Diagn Interv Radiol 2020; 26: 315–22. doi: 10.5152/dir.2020.20351 32558646 PMC7360076

